# Emphysematous Pyelonephritis: A single center review

**DOI:** 10.12669/pjms.36.ICON-Suppl.1728

**Published:** 2020-01

**Authors:** Albeerdy Mohammad Irfaan, Nadeem Ahmed Shaikh, Anila Jamshaid, Abdul Hafeez Qureshi

**Affiliations:** 1 Albeerdy Mohammad Irfaan, MBBS. Resident, Urology Department, The Indus Hospital, Karachi, Pakistan; 2 Nadeem Ahmed Shaikh, MBBS. Resident, Urology Department, The Indus Hospital, Karachi, Pakistan; 3 Dr. Anila Jamshaid, MBBS FCPS. Head of Department, Urology Department, The Indus Hospital, Karachi, Pakistan; 4 Abdul Hafeez Qureshi, MBBS, FCPS. Senior Consultant, Urology Department, The Indus Hospital, Karachi, Pakistan

**Keywords:** Emphysematous Pyelonephritis, Outcome, Mortality, Conservative management

## Abstract

**Background and Objective:**

Emphysematous pyelonephritis (EPN) is a rare, life-threatening necrotizing renal parenchymal infection. Traditional management of EPN with nephrectomy had a mortality of 40-50%. The purpose of this case series was to assess the management, biochemical factors, and outcome of EPN patients.

**Methods:**

In this retrospective study, patients admitted to The Indus Hospital, Karachi with a diagnosis of EPN from January 2010 to February 2019, were grouped according to the Huang Tseng Classification (HTC). Their biochemical parameters, sensorium states and outcomes were recorded and analysed.

**Results:**

Twenty patients were reviewed (9 males). No mortality was recorded. 11 patients (55%) were treated conservatively with only intravenous antibiotics and eight patients underwent an intervention: minimally invasive with drain placement in six patients, and invasive in four patients (two underwent subsequent nephrectomy, and two patients had nephrectomy only). One patient left against medical advice. All patients had decreased serum creatinine levels and total leucocyte counts on discharge.

**Conclusion:**

EPN can be successfully managed conservatively for HTC Grade-1 and 2. Conservative treatment may be tried in higher grades, but poor response should lead to prompt escalation of treatment.

## INTRODUCTION

Emphysematous pyelonephritis (EPN) is an acute severe necrotizing infection resulting in the presence of gas in the renal parenchyma, collecting system or perinephric tissue.^1^ The first reported case was in 1898 by Kelly and McCullum^1^ but the prevalence in Western countries remains low at 1-2 cases annually. Mortality is due to septic complications arising from the disease process.^1^ Diabetes mellitus (DM) is the most common associated factor^2^ as is the presence of urinary tract obstruction in 20-45%.^1^ There is a higher predilection for females.^3^,^4^ Two decades ago, nephrectomy was the standard treatment of care as non-surgical treatments led to mortality in 60-80% of patients.^5^ Computer Tomography (CT) imaging, as well as the advent of newer and better antibiotics, combined with an ever evolving multidisciplinary care, particularly in the critical care management of sepsis has enabled an overall decline in mortality rates to 20-25%.^2^ This study was designed to assess our management of the disease and analyse any associations of outcome with presenting biochemical variables.

## METHODS

The electronic medical records were reviewed with the keywords ‘Pyelonephritis’ and ‘Emphysematous Pyelonephritis’ and patients with CT scans showing Emphysematous Pyelonephritis were selected. The CT scans were again reviewed for confirmation of the disease and its grading. Gender, age, presence of DM (raised Hba1c or known disease), CT grading, presence of urinary obstruction, serum creatinine, total leukocyte count (TLC), platelets at presentation and discharge, altered sensorium, septic shock, mode of treatment (medical therapy alone vs intervention) and outcome were recorded. All appropriate ethical approvals were in place (IRD_IRB_2019_03_003).

Initial diagnosis was recorded as acute pyelonephritis (APN), urosepsis or multi-organ dysfunction (MODS). APN was diagnosed by the triad of fever, loin pain and dysuria. Urosepsis was defined as pyuria with markers of sepsis syndrome and positive urinary and/or blood culture. MODS was defined as clinical or biochemical markers of dysfunction of two or more organ systems in the presence of sepsis. The diagnosis of EPN was confirmed by a CT scan of abdomen in all patients. Cases were classified based on radiographic findings described by Huang and Tseng.^4^ Group-A were of Grades-1 and 2 whilst group B included Grades-3 and 4.

All patients were treated by a multidisciplinary team comprising of the nephrology, urology and intensive care team if the patient was in the ICU along with necessary allied health professionals. Standard treatment protocols for sepsis such as fluid resuscitation, insulin therapy as well as antibiotic therapy were followed. Empirical antibiotic treatment was with renally adjusted doses of broad spectrum antibiotics (piperacillin/tazobactum or meropenem). Combinations of antibiotics was used in all patients as first line therapy and changed depending on the microbiological culture reports. The need for renal replacement therapy (RRT) was based on clinical and biochemical indication. Drain placement was performed when patients did not respond to initial measures. A nephrectomy was performed in those patients who did not improve after drain placement. Patient outcome was divided into conservative and non conservative. Non conservative management included minimally invasive drainage and nephrectomy.

Data was entered in SPSS version 21. Calculations of mean ± standard deviation for normal, continuous variables and frequency and percentage for categorical variables were recorded. Independent t-test or Fisher’s exact test was used to compare continuous variables and chi square test to compare categorical variables. P ≤ 0.05 was considered as significant.

## RESULTS

Twenty patients met keyword criteria and had CT confirmation of EPN and were included in the study. The mean age of patients was 47.3±13 years and 45% were males. There was no in-hospital mortality although the outcome in one patient who left against medical advice is unknown.

DM was found in 55% and 45% had urinary tract obstruction. About 45% patients presented with altered sensorium and 25% presented in shock. *E.coli* was grown in the urine of 80% of patients and 25% had *E.coli* in blood cultures. The median hospital stay was 12, with a minimum of three days and a maximum of 37 days. Further blood parameters are described in Table-I.

**Table I:** Descriptive analysis.

*Platelets(10^9^/L)*	
Mean±SD	350±177.5
Min-Max	85-703
*Creatine on admission (mg/dL)*	
Median(IQR)	1.9(1.4-3.2)
Min-Max	1-6.3
*Creatine on discharge(mg/dL)*	
Median(IQR)	1.1(0.8-1.5)
Min-Max	0.6-5.2
*Total leukocyte count on admission(10^9^/L)*	
Mean±SD	19.2±7.5
Min-Max	6.4-32.8
*Total leukocyte count on discharge(10^9^/L)*	
Mean±SD	8.8±2.9
Min-Max	4-14.9

**Table II:** Huang Tseng Classification.

	Description
Grade 1	Gas in the collecting system
Grade 2	Renal parenchymal gas without extension
Grade 3	Perinephric/Paranephric extension of gas
Grade 4	EPN Bilaterally or in a solitary kidney

**Table III:** Patient CT scan grades and outcomes.

CT scan grade n(%)	
1	8(40)
2	4(20)
3A	2(10)
3B	3(15)
4	2(10)
Not assessed	1(5)
*Treatment*	
Conservative	12(60)
Drain	3(15)
Drain+I&D	1(5)
Drain+nephrectomy	2(10)
Nephrectomy	2(10)
*Outcome*	
Discharged	19(95)
LAMA	1(5)

Eleven (91%) of patients with Grade-1 or 2, were treated conservatively, while 7 (87.5%) of patients with a higher grade, required some form of intervention. Patients had a mean TLC of 19.2±7x 10^9^/L on admission which reduced to a mean of 8.8±2.9x 10^9^/L on discharge. No statistically significant association was found between DM or urinary tract obstruction in relation to EPN. Likewise, there was no statistical significance between the platelet count on admission v/s discharge. Conservative treatment of grade 1/2 was associated with a good recovery, with a mean hospital stay of 9.2 days as compared to 19 days for non conservative. There was a statistically significant decrease in the serum creatinine and TLC on admission compared to that at discharge.

## DISCUSSION

EPN is a severe, necrotizing infection characterized by bacterial production of gas within the renal parenchyma. The conditions required for the generation of EPN are a) the presence of pathogenic bacteria capable of mixed acid fermentation,^6^ b) high levels of glucose in tissue, c) impaired tissue perfusion.^7^ These factors collectively work to rapidly progress the disease process. DM and urinary tract obstruction have been implicated with EPN.^8^ However, concurring with Eswarappa et al,^9^ we did not find any significant relationship with either.

Some factors have been associated with increased risk in mortality in EPN, including thrombocytopenia, altered sensorium and/or shock at initial presentation along with polymicrobial infections.^10^,^11^ We observed that patients in shock, low platelet count and/or a serum creatinine greater than 3.3 mg/dl subsequently required non-conservative treatment. This is consistent with Wan YL *et al’s* observations that patients with higher creatinine levels (>1.4 mg/dl) and thrombocytopenia (<60,000/mm3) were at high risk for complications^12^ 80% of our patients had E coli in their urine- a trend which has been seen in many studies where E coli was the most commonly identified organism in urine cultures.^10^,^13^-^15^

About 91% of patients who had Grade-1 and 2 EPN (Group A) were managed conservatively, which reflects recent studies where such grades have been successfully managed conservatively.^16^ However, the management of EPN requires an aggressive approach, and the clinical picture of the patient should be carefully monitored, and if needed, a step up in the management should not be delayed if patient does not respond to antibiotics alone.^16^ Only one patient in Group-B (Grade-3 and 4 EPN) was treated conservatively- reflecting some case reports where patients with Grade-3 and 4 have been treated with only antibiotics,^17^ with the rest of patients not responding initially to antibiotics alone, and needing either a drain placement or a nephrectomy before finally being stabilized and eventually discharged.

**Table IV:** Paired wise comparison.

	Mean±SD	Min-Max	Median(IQR)	P value
*Creatinine (mg/dL)*				
On admission	2.5±1.5	1-6.3	1.9(1.4-3.2)	0.001*^†^
On discharge	1.4±1.0	0.6-5.2	1.1(0.8-1.5)	
*Total leukocyte count (10^9^/L)*				
On admission	19.2±7.5	6.4-32.8	18.3(13.8-25.9)	0.000**^†^
On discharge	8.8±2.9	4-14.9	8.4(6.2-11.6)	

* P-value<0.05,

** P-value<0.0001;

† Paired t test,

† Wilcoxon test, μ drain placement/nephrectomy.

### Limitations of the study

It is a retrospective study with small sample size which limits statistical inference. However EPN is still a rare condition. Most studies have also reported fewer or similar patient numbers.^11^,^12^,^14^,^15^ Though more than one risk factor was identified in 45% of patients, prompt intervention coupled with antibiotic therapy and good supportive care improved patient outcomes. A gradual paradigm shift is taking place with aggressive supportive and conservative measures being employed, with satisfactory results. This must not however take precedence of the clinical course of the disease, and if the patient is not responding to conservative measures alone, prompt measures such as drain placement and ultimately nephrectomy should be taken to ensure the survival of the patient.

## CONCLUSION

Patients treated for emphysematous pyelonephritis had no mortality recorded in this series. Grades 1 and 2 were successfully treated conservatively whilst grades 3 and 4 warranted further procedures such as drain placement and nephrectomy.

### Authors’ Contribution

**AMI:** Compiled all the raw data, Writing up and Editing of the manuscript, Final version of manuscript. Is also responsible for integrity of research.

**NAS:** Statistical Analysis of data and Editing of manuscript.

**AJ and AHQ:** Management of patients (both medical and surgical) and Editing of manuscript.

## References

[1] Ubee SS, McGlynn L, Fordham M (2011). Emphysematous pyelonephritis. BJU Int.

[2] Falagas ME, Alexiou VG, Giannopoulou KP, Siempos II (2007). Risk factors for mortality in patients with emphysematous pyelonephritis:a meta-analysis. J Urol.

[3] Abdul-Halim H, Kehinde EO, Abdeen S, Lashin I, Al-Hunayan AA, Al-Awadi KA (2005). Severe emphysematous pyelonephritis in diabetic patients:diagnosis and aspects of surgical management. Urol Int.

[4] Huang JJ, Tseng CC (2000). Emphysematous pyelonephritis:clinicoradiological classification, management, prognosis, and pathogenesis. Arch Intern Med.

[5] Hooton TM, Bradley SF, Cardenas DD, Colgan R, Geerlings SE, Rice JC (2010). Diagnosis, prevention, and treatment of catheter-associated urinary tract infection in adults:2009 International Clinical Practice Guidelines from the Infectious Diseases Society of America. Clin Infect Dis.

[6] Turney JH (2000). Renal conservation for gas-forming infections. Lancet.

[7] Vivek V, Panda A, Devasia A (2012). Emphysematous pyelonephritis in a renal transplant recipient--is it possible to salvage the graft?. Ann Transplant.

[8] Pontin AR, Barnes RD (2009). Current management of emphysematous pyelonephritis. Nat Rev Urol.

[9] Eswarappa M, Suryadevara S, John MM, Kumar M, Reddy SB, Suhail M (2018). Emphysematous Pyelonephritis Case Series From South India. Kidney Int Rep.

[10] Misgar RA, Mubarik I, Wani AI, Bashir MI, Ramzan M, Laway BA (2016). Emphysematous pyelonephritis:A 10-year experience with 26 cases. Indian J Endocrinol Metab.

[11] Kapoor R, Muruganandham K, Gulia AK, Singla M, Agrawal S, Mandhani A (2010). Predictive factors for mortality and need for nephrectomy in patients with emphysematous pyelonephritis. BJU Int.

[12] Wan YL, Lo SK, Bullard MJ, Chang PL, Lee TY (1998). Predictors of outcome in emphysematous pyelonephritis. J Urol.

[13] Torres H, Sharma P (2018). Bilateral emphysematous pyelonephritis. Urol Case Rep.

[14] Shokeir AA, El-Azab M, Mohsen T, El-Diasty T (1997). Emphysematous pyelonephritis:a 15-year experience with 20 cases. Urology.

[15] Uruc F, Yuksel OH, Sahin A, Urkmez A, Yildirim C, Verit A (2015). Emphysematous pyelonephritis:Our experience in managing these cases. Can Urol Assoc J.

[16] Jain A, Manikandan R, Dorairajan LN, Sreenivasan SK, Bokka S (2019). Emphysematous pyelonephritis:Does a standard management algorithm and a prognostic scoring model optimize patient outcomes?. Urol Ann.

[17] Mousa R, Alshamsi H, Eid K, Malalla B, Abandi A, Naami A (2018). Emphysematous pyelonephritis treated successfully with conservative management:A case report. Int J Case Rep Imag.

